# Primary cardiac tumors on the verge of oblivion: a European experience over 15 years

**DOI:** 10.1186/s13019-015-0255-4

**Published:** 2015-04-18

**Authors:** Andreas Habertheuer, Günther Laufer, Dominik Wiedemann, Martin Andreas, Marek Ehrlich, Claus Rath, Alfred Kocher

**Affiliations:** Department of Cardiac Surgery, Vienna General Hospital, Medical University of Vienna, Waehringer Guertel 18-20, A-1090 Vienna, Austria

**Keywords:** Primary cardiac tumors

## Abstract

**Background:**

Primary tumors of the heart represent an exceedingly rare entity in cardiac surgery and literature regarding management and outcome is rare. The aim of this study was to translate 15 years of experience in both multimodal diagnosis and surgical treatment of one of the largest collective of patients in literature into a detailed analysis of patient prognosis, mean survival and best treatment approach.

**Methods and results:**

All patients who underwent open-heart surgery at the Hospital of the Medical University of Vienna for primary cardiac tumor excision between 1999 and 2014 were analyzed retrospectively. Mean follow-up was 76.8 months. Descriptive statistical measurements were applied.

113 patients were identified, 71 (62.8%) female and 42 (37.2%) male patients with a mean age of 57.9 ± 16.8 years. 90.3% (n = 102) masses were benign, 9.7% (n = 11) were malignant. Complete resection was possible for 99% and for 18.2% of benign and malignant masses, respectively. 2.9% of benign tumors and 45.5% of malignant tumors relapsed. The 30-day mortality was 1.8% (n = 2). Mean survival was 187.2 ± 2.7 months and 26.2 ± 9.8 months for benign and malignant pathologies, respectively. Sarcoma patients who underwent adjuvant combination-chemotherapy or adjuvant mono-chemotherapy and radiation had a statistically significant survival advantage of 41.5 months.

**Conclusion:**

Primary cardiac tumors remain challenging in the clinical setting. A multimodality treatment approach especially for sarcoma patients prolongs mean survival and should be regarded as the standard of care.

## Background

Cardiac tumors, both benign and malignant represent extremely rare diseases and literature on both management and outcome is quite limited. Based upon the data of 22 large autopsy series reported by McAllister et al., the frequency of primary cardiac tumors is approximately 0.02%, corresponding to 200 tumors in 1 million autopsies [1,2]. Malignant tumors metastatic to the heart outnumber primary malignant cardiac tumors by at least a 30-to-1 ratio in the majority of autopsy series [3]. The reported incidence of primary cardiac tumors among the general population has varied between 0.001% and 0.03% in most studies with cardiac tumors representing only 0.3% of all open-heart surgeries [4].

To our knowledge, the first reports on intracavitary mass formation of the heart are to be traced back to Belgium in 1685 when Zollicoffer wrote “de polypo cordis”. Bahnson and Newman [5] performed the first successful open surgical excision of a primary cardiac tumor using the inflow obstruction technique in 1952 and only 2 years later Crafoord et al. reported the first successful atrial myxoma excision using a heart lung bypass [6].

Unless obstructing intracardial flow or interfering with the valvular [7] and conduction system respectively, cardiac neoplasms do have the potential to remain clinically silent until they reach an advanced stage, thereby limiting therapeutic options especially for those with malignant transformations [8]. The majority of primary cardiac tumors are benign with more than 80% being myxomas in various locations and dyspnea being the most common reason for initial clinical consultation [9]. Malignant cardiac tumors are mainly comprised of various subsets of sarcomas with primary cardiac lymphomas forming only a small fraction [2,10-13]. The clinical presentation of patients suffering from malignant transformations is usually more severe.

The overall objective of the present study was an in-depth characterization of both benign and malignant primary cardiac tumors, their frequency as well as age and gender distribution. 15 years of combined experience in both diagnosis and multimodal treatment of one of the largest collective of patients in the scientific literature were translated into a detailed analysis of patient prognosis, mean survival and risk of tumor relapse matched to the corresponding neoplastic etiology using descriptive statistical measurements.

## Methods

The Medical University of Vienna Medical Record system and the patient database of the Department of Cardiac Surgery were retrospectively reviewed to identify patients with primary cardiac neoplasias. A 15-year period between 1999 through 2014 was analyzed. Approval to conduct this study was obtained by the Institutional Review Board of the Medical University of Vienna and patient consent was waived. The medical records of all patients with primary heart tumors were reviewed and details regarding presentation, diagnosis, treatment, and follow-up were obtained. Pathology reports were used to definitely evaluate both dignity and etiology. Patients with tumors metastatic to the heart and those in whom a primary cardiac origin was in doubt were excluded. 2 patients with suspected primary benign cardiac tumors were subsequently excluded from the study as their tumors proved to be echinococcal cysts.

Furthermore, a telephone follow-up was performed for every patient. Detailed information was requested from local healthcare providers if necessary. Survival data were completed with the nationwide statistical database (‘Statistics Austria’), in which every death is recorded. The follow-up time was calculated either to death or to the last verified contact with the living patient. Furthermore, tumor-related events were assessed in the medical records and through the telephone follow-up. Median follow-up was 76.8 months (0–180 months).

Treatment consisted of open or minimally invasive heart surgery for treatment of benign tumors and open-heart surgery followed by adjuvant therapy for malignant tumors. Multiple groups of different adjuvant regimens could be identified. Adjuvant therapy for sarcoma patients consisted of either combination chemotherapy (6 courses of ifosfamide (1500 mg/m^2^, days 1–4), dacarbazine (200 mg/m^2^, days 1–4) and doxorubicin (25 mg/m^2^, days 1–2) administered in 14-day intervals and granulocyte-colony stimulating factor (30 × 10^6^ IU/day, s.c.) on days 5–13) or 6 courses of mono-chemotherapy (doxorubicin) and radiation, or 6 courses of mono-chemotherapy alone (doxorubicin or herceptin), or radiation alone. One sarcoma patient did not receive any adjuvant therapy. Radiation dose was age, gender and seize adjusted and ranged from a total dose of 15 Gy to 60 Gy. Adjuvant therapy for our patient suffering from a non-Hodgkin lymphoma (diffuse large B-cell lymphoma) consisted of 6 courses of rituximab, cyclophosphamide, doxorubicin, vincristine and prednisone (R-CHOP).

## Statistical analysis

Continuous data are presented as means ± standard deviation; skewed data are presented as median and interquartile range. The Kolmogorov–Smirnov test was used to check for distribution before any additional analysis was conducted. Categorical data are presented as percentage. Differences between groups were compared using Pearson’s Chi square test for categorical variables and Student’s T test for continuous variables. Long-term survival of groups was evaluated and compared using the Kaplan Meier Survival plot and the log-rank test, respectively. The number of patients at risk was assessed using life tables. All reported P values are 2-sided and are considered statistically significant and highly statistically significant if below 0,05 and below 0.01, respectively. All statistical analyses were performed using SPSS 21.0 (IBM, Armonk, NY, USA).

## Results

### Patient population

The baseline characteristics of patients are depicted in Table 1. A total of 113 patients underwent surgery for primary cardiac tumor excision within the study period. Taking all tumor etiologies into account women suffering from a cardiac tumor were statistically significant older (p = 0.02) than their male counterparts at the time of clinical presentation (61.6 ± 14.5 years vs. 51.6 ± 18.7 years). Analysis for age distribution revealed two peaks at 50 years and 65 years, respectively. Women had the highest risk of cardiac tumor formation at 55 and 65 years and men had the highest risk at 50 and 65 years, respectively.

**Table 1:** **Baseline patient characteristics**

Parameter	Benign (n = 102)	Malignant (n = 11)	P value
Age	58.9 ± 16.4	48.8 ± 18.7	0.059
Female gender	66 (64.7)	5 (45.5)	0.209
Urgent intervention	22 (21.6)	10 (90.9)	**<0.01**
Coronary artery disease	8 (7.8)	0 (0.0)	0.335
Valvular heart disease	12 (11.8)	0 (0.0)	0.229
Congenital heart disease	0 (0.0)	1 (9.1)	0.742
Thromboembolic disease	3 (2.9)	0 (0.0)	0.564

Data presented as mean ± standard deviation, n (%) or mean (range).

### Patient presentation

The picture of patients with benign cardiac tumors was distinct from that of malignant transformation. Common findings included symptoms of congestive heart failure (CHF) such as dyspnea (27.5%, n = 28) and central thromboembolism (8.8%, n = 9), including basilar artery thromboembolism. It was not uncommon for some patients to have undergone medical treatment for CHF before the diagnosis of a cardiac tumor as its underlying etiology was made. 26.5% (n = 27) of benign masses were incidental findings during medical evaluations performed for other reasons (70.4%, n = 19 were actually evaluated for other cardiac interventions). Only 8 patients with benign masses (7.8%) were truly asymptomatic. Of note, one patient presented with paroxysmal symptoms of catecholamine excess and was found to have a hormone secreting left atrial paraganglioma linked to a succinate dehydrogenase mutation.

The clinical presentation of patients with an underlying malignant disease was more severe with 27.7% reporting syncope as initial disease presentation and 18.2% presenting with cardiac arrest and under mechanical resuscitation. Of note, one patient developed an obstructive left atrial pleomorphic sarcoma 8 years upon heart transplantation in his cardiac allograft. He died within 2 weeks after the onset of initial symptoms including dyspnea due to cardiac arrest. Our non-Hodgkin patient initially presented with palpitations and arrhythmias, however no structural abnormalities could be detected on echocardiography. Within a period of only two weeks she developed progressive dyspnea, facial edema and facial erythema due to almost completely obstructive masses filling the right atrium and right ventricle (superior vena cava syndrome) [2].

### Tumor characteristics

Tumor characteristics are summarized in Tables 2 and 3, respectively. The majority of cases (90.3%) were benign transformations. Myxomas of the left atrium (76.5%) and fibroelastomas (12.7%) of various locations were largely predominant. The localization of fibroelastomas was more heterogenous with a high affinity for aortic and mitral valve leaflets. In general, myxomas were large obtructive masses measuring up to a couple of centimeters while fibroelastomas appeared as small vegetation-like lesions on valvular leaflets and closely resembled infectious or thrombotic valvular vegetations on echocardiography. Both left sided myxomas and fibroelastomas had a tendency for central nervous system and peripheral embolization (11.5% vs. 15.4%). Malignant cardiac tumors were mainly comprised of various subsets of sarcomas (90.9%) with primary cardiac lymphomas forming only a small fraction. Non-Hodgkin lymphomas specifically diffuse large B-cell lymphomas accounted for less than 10%. The most common sarcoma subtype found in our study was a highly malignant angiosarcoma (n = 3).

**Table 2:** **Characteristics of primary benign cardiac tumors**

Histology and location	No. of patients (% of benign)	Tumor relapse	Mean survival (months)
*Benign*	*102 (90.3%)*	*3*	187.2 ± 2.7
Myxoma	78 (76.5%)	3	188.4 ± 2.5
Left atrium	68 (66.6%)		
Right atrium	8 (7.8%)		
Information not available	2 (2.0%)		
Lipomatous hypertrophy	1 (1.0%)	0	
right atrial roof and superior caval vein			
True lipoma	1 (1.0%)	0	
right atrial septum, obstructing right atrium			
Glomangioma	1 (1.0%)	0	
extracardial, between left atrium and pulmonary artery			
Hemangioma	2 (2.0%)	0	
left atrium, anterior mitral valve leaflet	1 (1.0%)		
right intralatrial septum	1 (1.0%)		
Fibroelastoma	13 (12.7%)	0	
pulmonary valve	1 (1.0%)		
aortic valve leaflets	6 (5.9%)		
mitral valve leaflets	2 (2.0%)		
papillary muscle	2 (2.0%)		
apex, occluding the left ventricle*	1 (1.0%)		
left ventricular outflow tract (LVOT)	1 (1.0%)		
Hamartoma	2 (2.0%)	0	68.5 ± 48.4
right atrium, completely obstructing	1 (1.0%)		
right ventricle, completely obstructing	1 (1.0%)		
hereditary paraganglioma (left atrium)**	1 (0.9%)	0	

*Data presented as mean ± standard deviation, n (%) or mean (range).*

*Patient underwent successful heart transplantation 4 months later.

**The tumor was hormone secreting with pheochromocytoma-like symptoms. On genetic evaluation patient was found to have a succinate dehydrogenase mutation.

**Table 3:** **Characteristics of primary malignant cardiac tumors**

Histology and location	No. of patients (% of malignant)	Tumor relapse	Distant metastasis	Mean survival (months)
*Malignant*	*11 (9.7%)*	*5*	9	26.2 ± 9.8
Sarcoma (all), subtypes	10 (91.0%), subtypes:	5	8	21.1 ± 10.7
Angiosarcoma	3 (27.3%)	2	3	25.3 ± 17.6
right atrium				
Intima sarcoma***	1 (9.1%)	0	1	1.0 ± 0.0
left atrium and anterior mitral valve leaflet, obstructive				
Spindle cell sarcoma	1 (9.1%)	1	0	7.0 ± 0.0
right atrium and SVC, completely obstructive				
Synovial sarcoma	1 (9.1%)	1	1	64.00 ± 0.0
right atrium/ventricle, obstructing SVC/IVC				
Pleomorphic sarcoma****	1 (9.1%)	0	1	0.0 ± 0.0
left atrium, completely obstructive				
Myxofibrosarcoma	2 (18.2%)	0	1	7.0 ± 0.0
left atrium				
Myeloid sarcoma	1 (9.1%)	1	1	
right atrium, obstructing superior and inferior caval vein				
NHL (DLCBL)******	1 (9.1%)	0	1	
right atrium and right ventricle, obstructive				

*Data presented as mean ± standard deviation, n (%) or mean (range). SVC, superior vana cava; IVC, inferior vena cava.*

***Patient underwent cardial autotransplantation.

****Patient had a heart transplantation 8 years ealier. Obstructing sarcoma formation in the left atrium of the heart allograft.

*****One Patient (0.9%) with left atrial myxoma had a tumor relapse as Myxofibrosarcoma 3 months upon surgery.

******NHL indicates Non Hodgkin Lymphoma of the Diffuse Large B-Cell Lymphpma type.

**Table 4:** **Postoperative parameters**

Parameter	Benign	Malignant	P value
Early mortality (30d)	1 (1.0)	1 (9.1)	0.053
Tumor related	1 (1.0)	1 (9.1)	0.053
Late mortality (>30d)	1 (1.0)	7 (63.3)	<0.01
Tumor related	1 (1.0)	7 (63.3)	<0.01
Mean survival (months)	187.2 ± 2.7	26.2 ± 9.8	<0.01
PM dependency	7 (6.9)	2 (18.2)	1.88
Tumor relapse	3 (2.9)	5 (45.5)	<0.01

Data presented as mean ± standard deviation, n (%) or mean (range).

Complete tumor excision was possible for 99% versus 18.2% of benign and malignant tumors, respectively.

### Tumor relapse and distant metastasis formation

Myxomas appeared to be the only benign masses with a tendency to recur (2.9%, n = 3). Of note, one myxoma relapsed as a myxofibrosarcoma and presented with indolent fever as the sole symptom highlighting the fact that those myxoma-myxofibrosarcoma transformations are seldom but do occur with a frequency of about 1.3%. Despite multimodality treatment of malignant tumors with surgical excision and chemotherapy there was a high rate of both tumor recurrence and distant metastasis development. 45.5% (n = 5) relapsed (p < 0.01) and 72.7% (n = 8) developed distant metastasis within the follow-up period.

### Mortality

Postoperative parameters are depicted in Table 4. The 30-day mortality of all causes was only 1.8% (2 of 113) despite the aggressive nature especially of sarcomas and the critical conditions in which patients with large obstructing masses were presenting, highlighting the importance of our center as one of most experienced in Europe.

**Figure 1:**
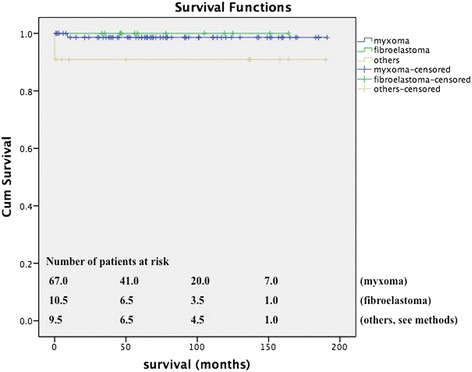
**Kaplan Meier showing mean survival of all patients with benign cardiac masses.** Mean survival = 187.2 ± 2.7 months.

**Figure 2:**
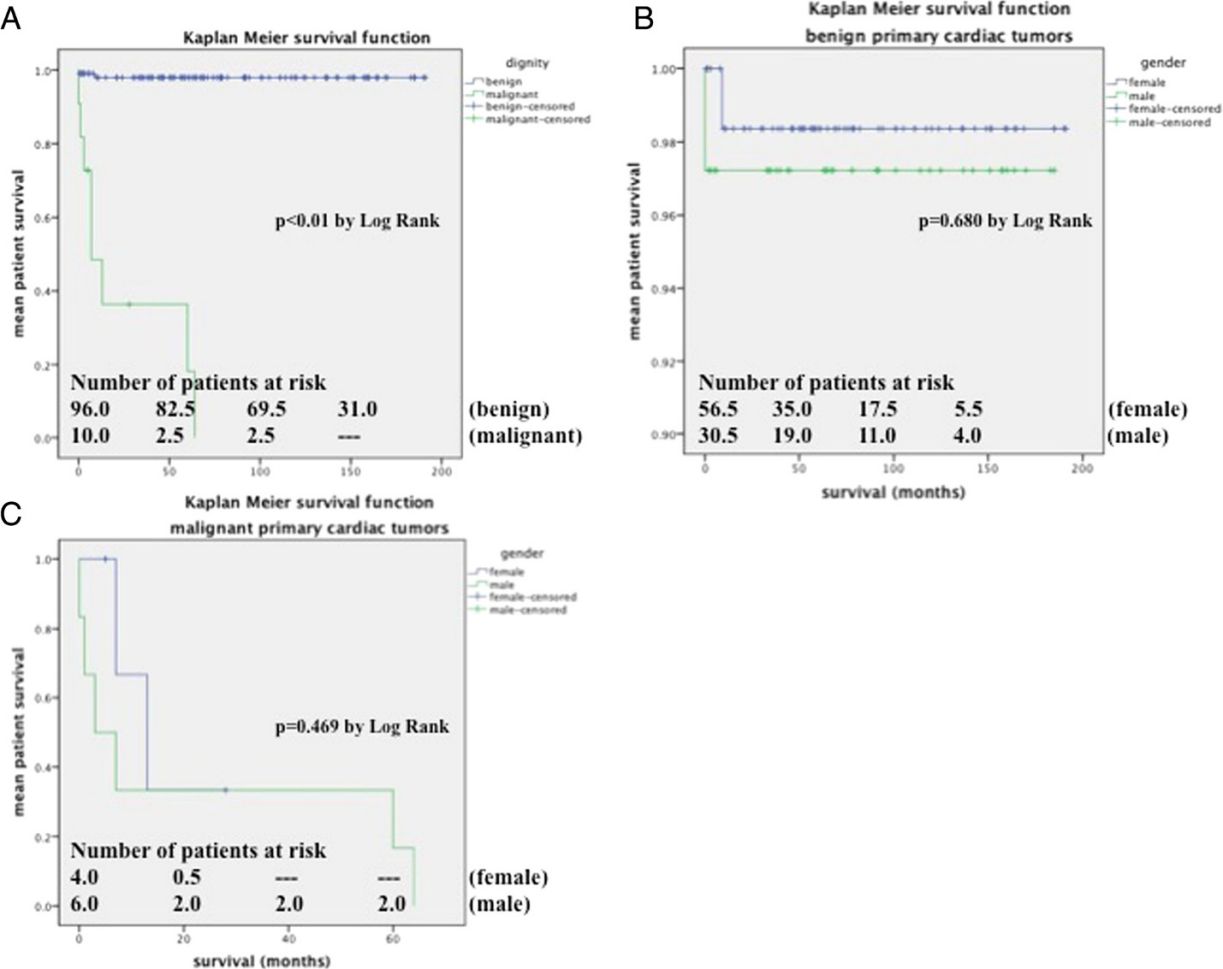
**(A) Mean survival for dignity matched groups (187.2 ± 2.7 months vs. 26.2 ± 9.8 months for benign versus malignant pathologies, p < 0.01). (B)** Mean survival for gender matched groups with benign pathologies (188.0 ± 2.9 months versus 179.9 ± 5.1 months for female versus male gender). **(C)** Mean survival for gender matched groups with malignant pathologies (16.0 ± 5.1 months versus 22.5 ± 12.5 months for female versus male gender).

The mean survival of patients with an underlying benign tumor was 187.2 ± 2.7 months compared to a mean survival of 26.2 ± 9.8 months in patients with a malignant tumor (Figures 1 and 2A). This finding was statistically significant by log rank (p < 0.01). When the study endpoint was reached, 98.0% of patients with benign masses were still alive compared to only 27.3% with malignant masses. The degree of technically feasible tumor resection had a significant effect on mean patient survival following surgery. The mean survival of patients in whom a complete resection was possible was 45.7 ± 16.4 months compared to only 9.3 ± 4.2 months for patients in whom the tumor could not be excised in-toto (p < 0.05 by log rank). Despite the fact that there was a female predominance regarding benign cardiac tumors, women had a statistically insignificant survival advantage of 10 months (188.0 ± 2.9 months versus 179.9 ± 5.1 months, p = 0.680, Figure 2B). The exact opposite applied to patients with an underlying malignant pathology where women had a statistically insignificant survival disadvantage (16,0 ± 5.1 months versus 22.5 ± 12.5 months, p = 0.469, Figure 2C). Among the sarcoma group, myeloid sarcomas had the best prognosis (patient alive at study endpoint) followed by synovial sarcomas (mean survival of 64.00 ± 0.0 months), angiosarcomas (25.3 ± 17.6 months), spindle cell sarcomas and myxofibrosarcomas (both 7.0 ± 0.0 months) and pleomorphic sarcomas (0.0 ± 0.0 months). This finding was statistically significant by log rank test (p < 0.05). Thus, the histologic sarcoma subtype appeared to have a major affect on the mean patient survival.

**Figure 3:**
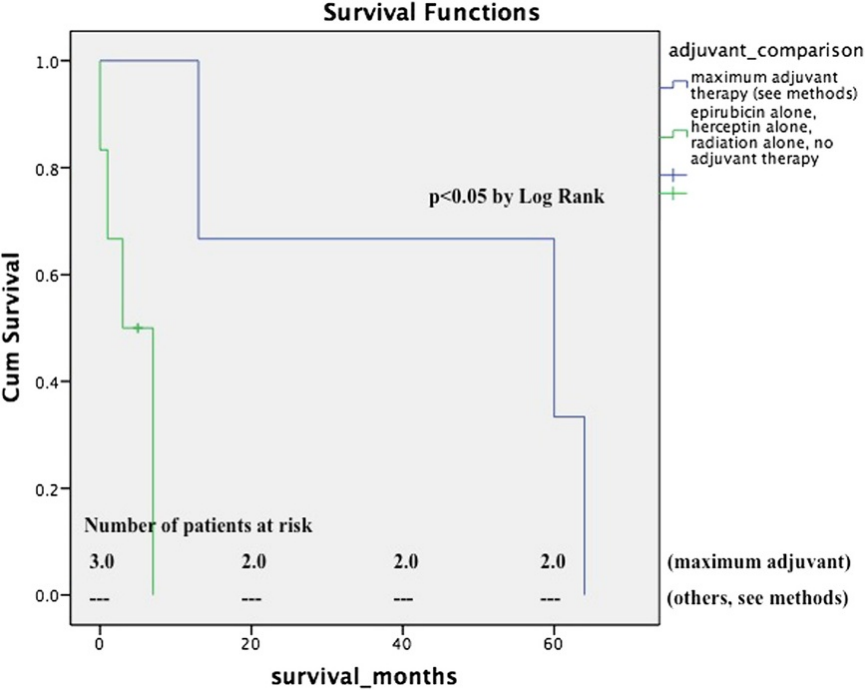
**Survival in comparison to adjuvant treatment regimen matched population.** Patients who were treated with adjuvant combination chemotherapy or doxorubicine and radiation had a statistically significant survival advantage (mean survival 45.7 ± 16.4 months) compared to patients who were treated with either radiation alone (4 months) or mono-chemotherapy (5 months) or who did not receive any adjuvant therapy. P < 0.05 by log rank.

In contrast to other authors [14] adjuvant therapy was found to have a favorable effect on mean patient survival. We compared the outcomes of various sarcoma adjuvant regimens consisting of either combination chemotherapy or 6 courses of doxorubicin and radiation, or mono-chemotherapy or radiation alone. Patients who underwent adjuvant combination chemotherapy or doxorubicin and radiation had a mean survival of 45.7 ± 16.4 months compared to 4.2 ± 1.4 months in patients who were treated with either radiation alone (median survival was 4 months) or mono-chemotherapy (median survival was 5 months) or who did not receive any adjuvant therapy. Statistical significance was reached by log rank (P < 0.05, Figure 3).

## Discussion

Taken together our results confirm previous reports in literature regarding the low incidence of primary cardiac neoplasias and the 9:1 distribution of benign tumors compared to malignant tumors. Primary cardiac tumors remain a rare entity and few surgeons worldwide have extensive experience in managing them. Secondary cardiac tumors are 20 to 40 times more common than primary tumors. The majority of cardiac tumors are metastatic, with the most common primary tumors being lymphomas, leukemias, malignant melanomas, lung and breast cancers [8,15,16].

The total number of primary cardiac tumors seen at our institution in 15 years was 113. These are thus exceedingly rare diagnoses, even in a tertiary center such as ours. Patients with a malignant mass are 8.6 years younger (mean 50.1 ± 19.1 years). Women diagnosed with a cardiac mass are 10 years older than their male counterparts (61.6 ± 14.5 years vs. 51.6 ± 18.7 years). There is a 2-peaked age distribution with women having the highest risk of cardiac tumor formation between at 55 and 65 years of age and men having the highest risk at 50 and 65 years, respectively. In contrast to benign cardiac tumors with a female predominance of 1.7:1, malignant tumors appear to be equally distributed between females and males. Benign cardiac tumors do have a predilection for left sided cardiac structures with myxomas sharing their most common origin from left atrial structures [9] and fibroelastomas having their most common origin at mitral valve leaflets or aortic valve leaflets. In contrast, malignant cardiac tumors have a predilection for the right side of the heart.

Both benign and malignant cases can be operatively managed with excellent results and low early postoperative mortality (30d). However, there is a significant difference in favor of benign tumors on midterm results and a difference in favor of adjunctive therapy on malignant patients and there appears to be some improvement in the current age of advancements in surgery and medical therapy, prolonging mean survival of patients with malignant cardiac tumors. There appears to be some recent advances in our center, mildely prolonging mean patient survival. The mean survival as reported in prior studies ranged from 6 months [10,17] to 18 months [8,13]. In the current series of patients in our center, the mean survival was 26 months, probably owing to our surgical expertise and the prompt multimodality treatment approach of combined early chemotherapy and radiation. Yet, the long-term survival of patients with malignant cardiac tumors remains poor and by far lacks behind the mean survival of 187 months (15.5 years) of patients with benign etiologies.

In literature the optimal treatment approach regarding both surgery and postoperative adjuvant therapy for these cancers remains unclear. Some earlier studies have demonstrated a better survival for patients who underwent a complete resection compared with patients who did not while others failed to reach statistical significance [12]. In the current study patients with a complete resection had a median survival of 45.7 ± 16.4 months compared to only 9.3 ± 4.2 months in those in whom a complete resection was not technically feasible. Therefore, a complete resection should always be the mainstay of surgical treatment. Regarding adjuvant tumor therapy Llombart-Cussac et al. in their retrospective review of 15 patients undergoing adjuvant chemotherapy after surgery found that this approach failed to modify the natural course of the disease [8,14]. In the present study 10 patients underwent adjuvant therapy consisting of either combination chemotherapy, or mono-chemotherapy with radiation or mono-chemotherapy or radiation alone. Patients who underwent adjuvant combination chemotherapy or combined doxorubicin mono-chemotherapy and radiation had a statistically significant survival advantage (45.7 ± 16.4 months compared to 4.2 ± 1.4 months, p < 0.05), however, a larger collective of patients would be needed to derive firm conclusion. Based on our results optimal patient management consists of early and total surgical tumor excision followed by combination adjuvant chemotherapy or adjuvant mono-chemotherapy and radiation. This approach is highly recommended and should be regarded as the standard of care.

Patients with malignant neoplasias cannot be considered for heart transplantation as solid organ transplantations necessitate postoperative immunosuppression. This would favor secondary metastatic disease.

In conclusion, especially malignant cardiac tumors require a multimodality treatment approach. Complete tumor resection and adjuvant therapy should be attempted whenever feasible and are to be regarded as the gold standard of care.

## Abbreviations

CHF: Congestive heart failure
Gy: Gray (untit)
IU: International units
IVC: Inferior vena vava
R-CHOP: Rituximab, cyclophosphamide, doxorubicin, vincristine, prednisolone
SVC: Superior vena cava
